# Association between the maternal protein nutrition status during pregnancy and the risk of preterm birth

**DOI:** 10.1111/mcn.13043

**Published:** 2020-08-20

**Authors:** Ting Xiong, Yuanjue Wu, Li Huang, Xi Chen, Yu Zhang, Chunrong Zhong, Qin Gao, Miao Hong, Xingwen Hu, Xuefeng Yang, Nianhong Yang, Liping Hao

**Affiliations:** ^1^ Department of Nutrition and Food Hygiene, Hubei Key Laboratory of Food Nutrition and Safety and the Ministry of Education (MOE) Key Lab of Environment and Health, School of Public Health, Tongji Medical College Huazhong University of Science and Technology 13 Hangkong Road Wuhan Hubei 430030 China; ^2^ Hubei Maternal and Child Health Hospital Wuhan China

**Keywords:** cohort study, gestational duration, plasma total protein, pregnant women, preterm birth

## Abstract

We aimed to assess protein nutrition status during pregnancy by maternal plasma total protein (MTP) levels in urban pregnant women and to explore the association between the trimester‐specific MTP levels and risk of preterm birth (PTB).

A prospective design was conducted in 3,382 mother‐newborn pairs with the second‐trimester maternal MTP information and in 3,478 mother‐newborn pairs with the third‐trimester MTP information. Multiple Cox proportional hazard regression and multiple linear regression were used to analyse the associations between MTP levels and PTB risk as well as gestational duration, respectively.

Nearly all the second‐trimester MTP levels were within the clinical reference range, but more than 40% of the third‐trimester MTP levels were less than the lower limit of normal. No significant association was found between the second‐trimester MTP level and PTB risk. However, the adjusted hazard ratios (HRs) of PTB across increasing quartiles of the third‐trimester MTP levels were 1.00 (reference), 0.59 (0.36, 0.95), 0.35 (0.20, 0.60), and 0.32 (0.19, 0.53) (*p*_for trend_ < 0.001), respectively. Each standard deviations increment of the third‐trimester MTP was associated with increase of 0.13 weeks in gestational duration. Moreover, stratified analyses showed that the effects of third‐trimester MTP on PTB risk and gestational duration were stronger in pregnant women carrying female offspring than those carrying male offspring (*p*_for interaction_ < 0.05).

The third‐trimester MTP level was inversely associated with PTB risk and was positively associated with gestational duration. Improving third‐trimester MTP level may be helpful for preventing PTB.

**Key messages**

- Even in urban pregnant women, more than 40% of the third‐trimester maternal plasma total protein (MTP) levels were less than the lower limit of normal.
- The third‐trimester MTP level was inversely associated with preterm birth (PTB) risk and was positively associated with gestational duration.
- The effects of the third‐trimester MTP level on PTB risk and gestational duration were stronger in pregnant women carrying female offspring than those carrying male offspring.

## 1 INTRODUCTION

Preterm birth (PTB), defined by the World Health Organization (WHO) as birth before 37 weeks of gestation, is considered a major health problem worldwide for a long time (“The prevention of perinatal mortality and morbidity. Report of a WHO Expert Committee,” 1970). It is the leading cause of death among children under 5 years of age (Frey & Klebanoff, 2016; Liu et al., 2016; Moster, Lie, & Markestad, 2008). Moreover, infants born preterm have higher rates of other long‐term morbidities, including asthma (Tronnes, Wilcox, Lie, Markestad, & Moster, 2013), learning disabilities (Aarnoudse‐Moens, Weisglas‐Kuperus, van Goudoever, & Oosterlaan, 2009), attention deficit disorder (Delobel‐Ayoub et al., 2009), emotional problems (Hovi et al., 2007; Van Lieshout, Boyle, Saigal, Morrison, & Schmidt, 2015) and insulin resistance and hypertension (Blencowe et al., 2012; Rotteveel, van Weissenbruch, Twisk, & Delemarre‐Van de Waal, 2008), compared with those born at term. PTB also represents a staggering economic burden to society, with an estimated cost of 26 billion dollars every year in the United States alone (Hall & Greenberg, 2016; McCabe, Carrino, Russell, & Howse, 2014). Of greater concern, the global incidence rate of PTB is as high as 11.1% and is steadily increasing in almost all countries (Liu et al., 2016). However, the aetiologies of PTB are largely unknown (Frey & Klebanoff, 2016). Thus, a better understanding of the causes and identification of modifiable risk factors of PTB is urgently needed.

Proteins status have been perceived as crucial for foetal health in past decades (Blumfield & Collins, 2014; Blumfield, Hure, Macdonald‐Wicks, Smith, & Collins, 2012; Morisaki et al., 2018; Ota, Hori, Mori, Tobe‐Gai, & Farrar, 2015; Switkowski et al., 2016). However, few studies have focused on the association between the maternal protein status during pregnancy and the risk of PTB. In a recent Cochrane review study, two early studies found that pregnant women who received nutritional advice resulting in an increase in dietary protein intake had fewer PTB risk than control subjects (Ota et al., 2015). In the same Cochrane review study, however, six studies found no significant effects of protein supplementation on the PTB incidence as well as gestational duration. Nevertheless, a recent case–control study revealed a statistically significant lower maternal protein intake among women who delivered preterm offspring than among control women. The study also found that the maternal protein intake correlated positively with the gestational duration of the offspring (Awasthi, Chauhan, Pandey, Singh, & Singh, 2015). However, these previous studies mainly focused on the direct association between maternal protein intake/supplementation and PTB risk, which may be limited by intrinsic difficulties associated with accurate assessments of the protein intake and individual differences in digestion and absorption among different pregnant women. The plasma total protein level, a long‐established biological marker of the protein nutrition status in the body, may be a good alternative to maternal protein intake to investigate the association between the protein nutrition status and gestation duration. However, few studies have reported the maternal plasma total protein (MTP) level during pregnancy in recent years and the association between MTP level and PTB risk.

Within this context, we aimed to assess protein nutrition status by MTP levels in urban Chinese pregnant women and to prospectively determine the association between MTP levels during pregnancy (both in the second trimester and in the third trimester) and the risk of PTB using data from mother‐newborn pairs enrolled in a large prospective cohort study.

## 2 SUBJECTS AND METHODS

### 2.1 Study design and participants

The present study used data from the ongoing Tongji Maternal and Child Health Cohort (TMCHC) study. The TMCHC was a prospective cohort study that enrolled urban pregnant women at 8 to 16 gestational weeks who did not have communication problems from four maternity centres in Wuhan in central China, to investigate the impacts of maternal diet and other lifestyle factors before and/or during pregnancy on pregnancy outcomes. The study was approved by the Ethics Review Committee of Tongji Medical College, Huazhong University of Science and Technology (no. 201302). All study participants were informed of the study protocol and provided informed consent before inclusion.

**FIGURE 1:**
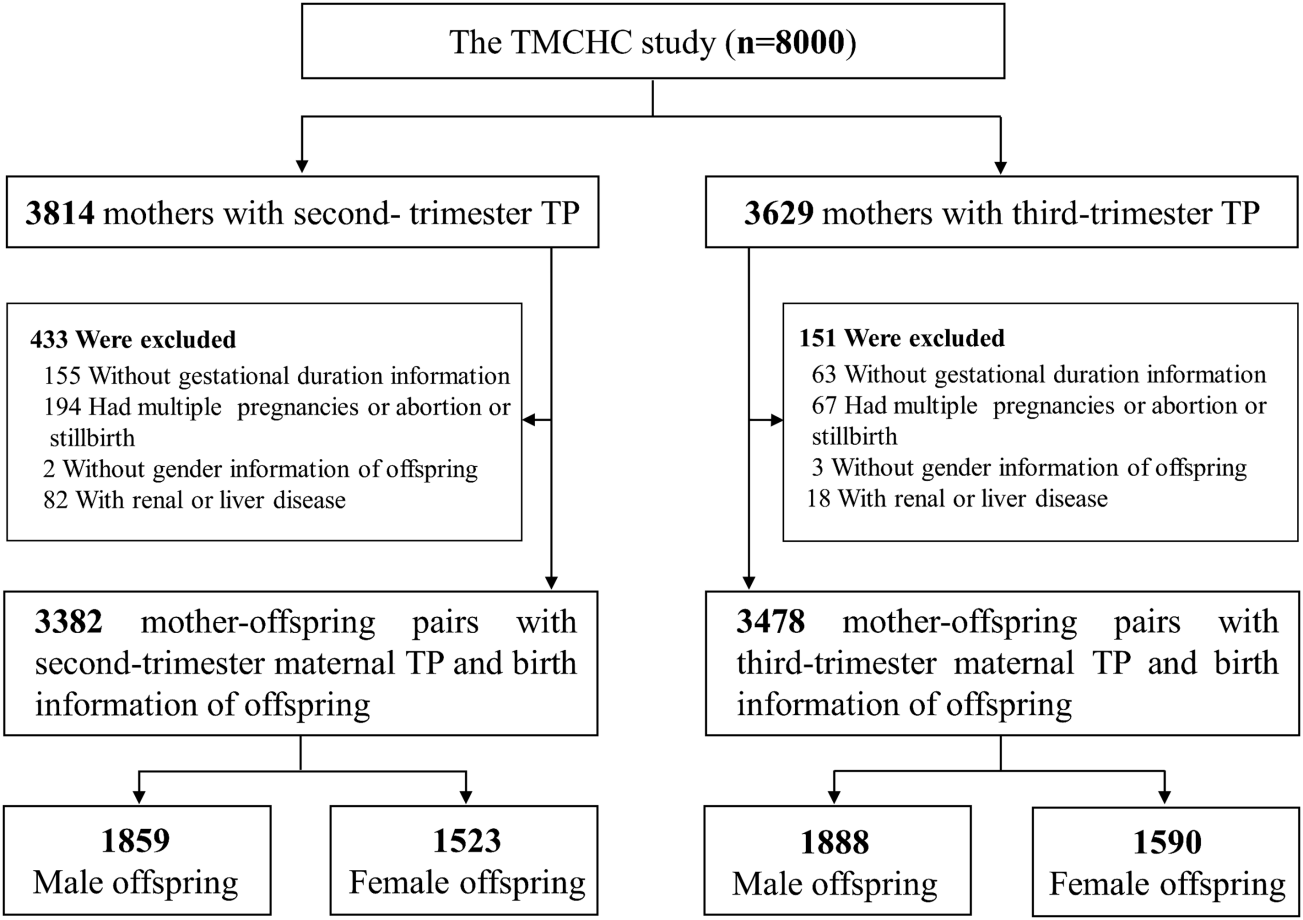
The flow chart of the participant selection process

From September 2013 to April 2016, 8,000 eligible pregnant women were invited and agreed to participate in the TMCHC study. Further detailed exclusion criteria and the corresponding numbers of excluded subjects are provided in Figure 1. Finally, 3,382 mother‐newborn pairs who underwent the maternal liver function test (LFT) during the second trimester and 3,478 mother‐newborn pairs who underwent maternal LFT during the third trimester were included in the current study.

### 2.2 MTP assessment

The data on MTP level (g/L) and other LFT parameters, such as alanine transaminase (ALT), aspartate transaminase (AST), alkaline phosphatase (ALP), γ‐glutamyl transferase (γ‐GT), total bilirubin (TBIL), direct bilirubin (DBIL) and indirect bilirubin (IBIL) levels, were obtained from the medical records collected from the regular antenatal examination performed by professional laboratory technicians. The local normal laboratory range of maternal TP during pregnancy ranged from 65 to 85 g/L.

### 2.3 Outcomes assessment

Information on neonatal birth outcomes, such as the date of birth, baby gender and delivery mode, was obtained from obstetric records. Gestational duration was calculated based on the self‐reported last menstrual period (LMP). When the pregnant women could not accurately report the LMP, we used the foetal crown‐lump length, which is measured in the first trimester using routine ultrasound examination, to calculate the gestational age at birth. Additionally, if the difference between the LMP‐based gestational age and crown‐lump length‐based gestational age exceeded 10 days, we chose the latter value (Hadlock, Shah, Kanon, & Lindsey, 1992). PTB was defined as delivery before 37 weeks of gestation (“The prevention of perinatal mortality and morbidity. Report of a WHO Expert Committee,” 1970).

### 2.4 Covariates assessment

Maternal characteristics, such as demographic and sociological characteristics, were recorded based on data from a structured questionnaire as a part of a face‐to‐face interview by well‐trained investigators upon enrolment. The questionnaire included recognized and some of putative confounding factors of PTB, such as maternal age, educational and income levels, parity, LMP, alcohol and cigarette abuse, presence of insomnia prior to the current pregnancy and menstrual periods and cycles. Educational levels were categorized as ≤9, 10–15 and ≥16 years of schooling completed. Income levels were categorized as ≤4,999, 5,000–9,999 and ≥10,000 Chinese Yuan (CNY) (1 CNY ≈ 0.13 EUR; 1 CNY ≈ $0.14 USD), according to the mean monthly household income per person. Smoking (or drinking) before pregnancy was defined as smoking (or drinking) no less than three times every week for more than 6 months before pregnancy. Otherwise, the status was defined as non‐smoking (or non‐drinking). Insomnia, which was the subjective feeling of pregnant women, was defined as difficulty initiating or maintaining sleep, or early awakening with inability to return to sleep, together with associated impairment of daytime functioning (Zhong et al., 2018). Gestational weeks at the time of LFT were calculated using the aforementioned methods based on LMP. Maternal height was measured using an automatic weight and height scale when the patient was barefoot. Information on gestational weight gain and gestational diabetes mellitus (GDM) were obtained from medical records.

### 2.5 Statistical analysis

All covariates were assessed for normality. Covariates which did not meet the criterion of normality, we transformed or analysed using non‐parametric methods. Descriptive statistics at baseline are presented as means [standard deviations (SDs)] for continuous variables and percentages for categorical variables. Subjects were divided into quartiles based on MTP levels. Basic characteristics were compared across MLP levels quartiles by analysis of variance test for continuous variables and *χ* ^2^ test for categorical variables.

The association of MTP level with PTB was evaluated by hazard ratios (HRs) with 95% confidence intervals (CIs) with multiple Cox proportional hazard regression. The mean value of each quartile of MTP levels were considered as continuous variable in the multiple Cox proportional hazard regression models to test for a linear trend. Association of MTP levels with gestation duration were estimated by multiple linear regression.

In model I, we adjusted for the following key confounders: maternal age at time of LFT, gestational weeks at time of LFT, gestational weight gain, prepregnancy weight, maternal height, menstrual period and cycle, caesarean delivery, GDM, baby gender, maternal educational and income levels, primiparity, and insomnia, drinking and smoking status before pregnancy. In model II, we further adjusted maternal serum levels of ALT, AST, ALP, γ‐GT, TBIL and IBIL based on model I. Model II was taken as the full model.

All statistical analyses were performed with SAS version 9.4 software (SAS Institute, Cary, NC, USA). Significance was set to two‐tailed *p* values <0.05 in all analyses.

## 3 RESULTS

### 3.1 Descriptive characteristics and maternal protein nutrition status during pregnancy

Table 1 describes mother‐offspring characteristics according to gestational stage and baby gender. On average, women underwent LFTs in the second trimester at a mean of 20.53 gestational weeks at a mean age of 28.63 years with a mean prepregnancy BMI of 20.77 kg/m^2^, and most of them (2,892, 85.51%) were nulliparous. Meanwhile, women underwent LFTs in the third trimester at a mean of 38.13 gestational weeks at a mean age of 28.50 years old with a mean prepregnancy BMI of 20.77 kg/m^3^, and most of them (2,888, 83.04%) were also nulliparous. Male offspring were born earlier and had a higher risk of PTB than female offspring.

**TABLE 1:** Characteristics of the mother‐offspring according to gestational stage and offspring gender^a^

	Second trimester				Third trimester			
	Total	Male offspring	Female offspring	*p* value	Total	Male offspring	Female offspring	*p* value
*N*	3,382	1,859	1,523		3,478	1,888	1,590	
Maternal age at LFT (years)	28.63 ± 3.37	28.68 ± 3.39	28.58 ± 3.35	0.435	28.50 ± 3.43	28.57 ± 3.43	28.42 ± 3.42	0.193
Prepregnancy weight (kg)	53.46 ± 7.58	53.52 ± 7.51	53.37 ± 7.66	0.568	53.44 ± 7.42	53.50 ± 7.29	53.36 ± 7.57	0.590
Maternal height (cm)	160.39 ± 5.02	160.49 ± 5.10	160.26 ± 4.93	0.173	160.38 ± 5.04	160.34 ± 5.13	160.43 ± 4.93	0.572
Prepregnancy BMI (kg/m3)	20.77 ± 2.67	20.77 ± 2.65	20.77 ± 2.70	0.987	20.77 ± 2.64	20.81 ± 2.60	20.73 ± 2.70	0.361
Gestational weight gain (kg)	16.00 ± 5.26	15.94 ± 5.37	16.08 ± 5.12	0.423	15.97 ± 4.89	15.86 ± 4.96	16.10 ± 4.80	0.159
Gestational week at LFTs (weeks)	20.53 ± 4.44	20.50 ± 4.43	20.56 ± 4.46	0.709	38.13 ± 3.38	38.10 ± 3.32	38.17 ± 3.44	0.514
Primiparity	2,892 (85.51%)	1,555 (83.65%)	1,337 (87.79%)	<0.001	2,888 (83.04%)	1,538 (81.46%)	1,350 (84.91%)	0.007
Caesarean delivery				0.326				0.042
Yes	1,254 (37.08%)	710 (38.19%)	544 (35.72%)		1,327 (38.15%)	756 (40.04%)	571 (35.91%)	
No	1,756 (51.92%)	950 (51.10%)	806 (52.92%)		2,020 (58.08%)	1,065 (56.41%)	955 (60.06%)	
Missing	372 (11.00%)	199 (10.70%)	173 (11.36%)		131 (3.77%)	67 (3.55%)	64 (4.03%)	
GDM				0.569				0.539
Yes	292 (8.63%)	154 (8.28%)	138 (9.06%)		343 (9.86%)	190 (10.06%)	153 (9.62%)	
No	2,954 (87.34%)	1,626 (87.47%)	1,328 (87.20%)		3,110 (89.42%)	1,687 (89.35%)	1,423 (89.50%)	
Missing	136 (4.02%)	79 (4.25%)	57 (3.74%)		25 (0.72%)	11 (0.58%)	14 (0.88%)	
Educational levels (years)				0.979				0.441
≤9	455 (13.45%)	252 (13.56%)	203 (13.33%)		410 (11.79%)	233 (12.34%)	177 (11.13%)	
10–15	887 (26.23%)	486 (26.14%)	401 (26.33%)		892 (25.65%)	473 (25.05%)	419 (26.35%)	
≥16	2,040 (60.32%)	1,121 (60.30%)	919 (60.34%)		2,176 (62.56%)	1,182 (62.61%)	994 (62.52%)	
Income levels (CNY^b^)				0.648				0.472
≤4,999	1,279 (37.82%)	716 (38.52%)	563 (36.97%)		1,231 (35.39%)	678 (35.91%)	553 (34.78%)	
5,000–9,999	1,488 (44.00%)	810 (43.57%)	678 (44.52%)		1,530 (43.99%)	835 (44.23%)	695 (43.71%)	
≥10,000	615 (18.18%)	333 (17.91%)	282 (18.52%)		717 (20.62%)	375 (19.86%)	342 (21.51%)	
Menstrual period (days)	5.70 ± 1.17	5.71 ± 1.15	5.69 ± 1.18	0.527	5.69 ± 1.19	5.72 ± 1.18	5.65 ± 1.21	0.081
Menstrual cycle (days)	30.40 ± 5.19	30.40 ± 4.99	30.40 ± 5.42	0.998	30.37 ± 4.32	30.38 ± 4.46	30.35 ± 4.14	0.865
Hepatitis B (yes)	0 (0.00%)	0 (0.00%)	0 (0.00%)	1.000	63 (1.81%)	32 (1.69%)	31 (1.95%)	0.575
Insomnia before pregnancy (yes)	1,332 (39.38%)	727 (39.11%)	605 (39.72%)	0.715	1,467 (42.19%)	798 (42.29%)	669 (42.08%)	0.899
Drinking before pregnancy (yes)	57 (1.69%)	25 (1.34%)	32 (2.10%)	0.089	50 (1.44%)	23 (1.22%)	27 (1.70%)	0.235
Smoking before pregnancy (yes)	115 (3.40%)	56 (3.01%)	59 (3.87%)	0.169	104 (2.99%)	50 (2.65%)	54 (3.40%)	0.197
**Biochemical parameters**								
ALT (U/L)	19.13 ± 15.79	18.80 ± 15.60	19.53 ± 16.01	0.115	11.61 ± 22.15	12.04 ± 23.32	11.10 ± 20.67	0.396
AST (U/L)	19.36 ± 9.10	19.14 ± 8.67	19.62 ± 9.60	0.299	16.90 ± 12.68	17.39 ± 15.70	16.32 ± 7.65	0.095
ALP (U/L)	51.48 ± 14.77	51.73 ± 15.64	51.18 ± 13.63	0.861	159.65 ± 65.63	164.75 ± 67.98	153.58 ± 62.19	<0.001
γ‐GT (U/L)	14.01 ± 8.22	14.03 ± 8.39	13.99 ± 8.01	0.539	12.47 ± 8.87	12.73 ± 9.29	12.17 ± 8.34	0.047
TBIL (umol/L)	6.28 ± 2.74	6.32 ± 2.89	6.24 ± 2.54	0.798	5.99 ± 3.24	5.98 ± 2.98	6.00 ± 3.53	0.851
DBIL (umol/L)	2.25 ± 1.04	2.24 ± 1.05	2.28 ± 1.03	0.165	2.42 ± 1.27	2.43 ± 1.41	2.41 ± 1.06	0.437
IBIL (umol/L)	4.02 ± 2.34	4.07 ± 2.43	3.96 ± 2.23	0.448	3.56 ± 2.74	3.59 ± 2.96	3.54 ± 2.45	0.693
MTP (g/L)	70.26 ± 3.75	70.27 ± 3.80	70.25 ± 3.69	0.896	65.95 ± 4.14	65.98 ± 4.23	65.91 ± 4.04	0.642
<65	255 (7.54%)	147 (7.91%)	108 (7.09%)	0.371	1,408 (40.48%)	766 (40.57%)	642 (40.38%)	0.907
65–85	3,127 (92.46%)	1,712 (92.09%)	1,415 (92.91%)		2,070 (59.52%)	1,122 (59.43%)	948 (59.62%)	
>85	0(0.00%)	0(0.00%)	0(0.00%)		0(0.00%)	0(0.00%)	0(0.00%)	
**Outcomes**								
Gestational duration (weeks)	39.50 ± 1.54	39.40 ± 1.55	39.62 ± 1.53	<0.001	39.59 ± 1.37	39.48 ± 1.44	39.72 ± 1.28	<0.001
PTB (%)	143 (4.23%)	92 (4.95%)	51 (3.35%)	0.021	120 (3.45%)	80 (4.24%)	40 (2.52%)	0.006

Abbreviations: ALT, alanine transaminase; AST, aspartate transaminase; ALP, alkaline phosphatase; BMI, body mass index; DBIL, direct bilirubin; GDM, gestational diabetes mellitus; IBIL, indirect bilirubin; LFT, liver function test; MTP, maternal plasma total protein; PTB, preterm birth; TBIL, total bilirubin; γ‐GT, γ‐glutamyl transferase.

^a^ Continuous variables were presented as mean (SD); categorical variables were showed as percentages (%).

^b^ CNY, Chinese Yuan, 1 CNY ≈ 0.13 EUR; 1 CNY ≈ 0.14 USD.

The maternal protein nutrition status evaluated by MTP level was also displayed in Table 1 **.** The mean value of second‐trimester MTP level was 70.26 ± 3.75 (g/L), and the mean value third‐trimester MTP level was 65.95 ± 4.14(g/L). Nearly all the second‐trimester MTP levels were within the clinical reference range, but more than 40% of the third‐trimester MTP levels were less than the lower limit of normal. Neither the second‐trimester MTP nor the third‐trimester MTP level exceeds the higher limit of normal.

The basic characteristics of the study subjects according to quartiles of the second‐trimester MTP and the third‐trimester MTP levels were displayed in Table S1 and Table S2, respectively.

### 3.2 Associations between the MTP level and the risk of PTB

No significant association between the second‐trimester MTP level and risk of PTB was observed (Table S3). However, Table 2 displayed that the risk of PTB decreased gradually with the increase in third‐trimester MTP. In the crude model, the HRs (95% CIs) of PTB across increasing quartiles of third‐trimester MTP were 1.00 (reference), 0.58 (0.36, 0.93), 0.35 (0.21, 0.60) and 0.41 (0.25, 0.67) (*p*_for trend_ < 0.001). The results were robust after adjusting for maternal age at the time of LFT, gestational weeks at the time of LFT, gestational weight gain, prepregnancy weight, maternal height, the menstrual period and cycle, caesarean delivery, GDM, baby gender, maternal educational and income levels, primiparity, insomnia, and the drinking and smoking status before pregnancy, and the corresponding HRs were 1.00 (reference), 0.58 (0.36, 0.93), 0.33 (0.19, 0.58) and 0.30 (0.18, 0.50) (*p*_for trend_ < 0.001). The results remained robust after further adjusting for ALT, AST, γ‐GT, ALP, TBIL and IBIL in model II, and the final HRs were 1.00 (reference), 0.59 (0.36, 0.95), 0.35 (0.20, 0.60) and 0.32 (0.19, 0.53) (*p*_for trend_ < 0.001). Furthermore, the risk of PTB for each SD increment of the third‐trimester MTP level was 0.67 (0.56, 0.81) in the full model.

**TABLE 2:** Association between third‐trimester MTP concentrations (g/L) and risk of PTB^a^

	Quartiles of third‐trimester MTP concentration, g/L				*p*_for trend_	Per SD increment of MTP
	Q1(48.0–63.1)	Q2(63.2–65.8)	Q3(65.9–68.5)	Q4(68.6–82.7)		
Crude model	1	0.58 (0.36, 0.93)	0.35 (0.21, 0.60)	0.41 (0.25, 0.67)	<0.001	0.70 (0.58, 0.84)
Model I	1	0.58 (0.36, 0.93)	0.33 (0.19, 0.58)	0.30 (0.18, 0.50)	<0.001	0.65 (0.54, 0.77)
Model II	1	0.59 (0.36, 0.95)	0.35 (0.20, 0.60)	0.32 (0.19, 0.53)	<0.001	0.67 (0.56, 0.81)

^a^ Model I were adjusted for maternal age, gestational weeks at time of LFT, gestational weight gain, prepregnancy weight, maternal height, menstrual period and cycle, caesarean delivery, GDM, baby gender, maternal educational and income levels, primiparity, and insomnia, drinking and smoking status before pregnancy based on crude model; model II were further adjusted for maternal serum ALT, AST, γ‐GT, ALP, TBIL, IBIL based on model I.

In stratified analyses, a significant sex‐specific association was found between the third‐trimester MTP level and the risk of PTB in our study (Table 3). In pregnant women with male offspring, the risk of PTB decreased gradually with the increase in third‐trimester MTP, similar to the total participants, and HRs of PTB across increasing quartiles of third‐trimester MTP levels were 1.00 (reference), 0.64 (0.34, 1.21), 0.43 (0.22, 0.84) and 0.49 (0.27, 0.91), respectively (*p*_for trend_ = 0.018) after adjustment for possible confounding factors in full model. In pregnant women with female offspring, the risk of PTB also decreased with the increase of third‐trimester MTP, and the odds ratios (ORs) of PTB across increasing quartiles of third‐trimester MTP levels were 1.00 (reference), 0.51 (0.23, 1.11), 0.17 (0.06, 0.50) and 0.10 (0.03, 0.31) (*p*_for trend_ < 0.001) in full model. However, it was obvious that the effects of third‐trimester MTP on PTB risk was stronger in pregnant women carrying female offspring than those carrying male offspring (*p*_for interaction_ = 0.003).

**TABLE 3:** Association between third‐trimester MTP (g/L) and PTB risk according to offspring gender^a^

Model	Male offspring	Female offspring	*p*_for interaction_
Crude model			0.013
Q1	1	1	
Q2	0.60 (0.33, 1.12)	0.55 (0.27, 1.15)	
Q3	0.48 (0.25, 0.91)	0.20 (0.07, 0.54)	
Q4	0.61 (0.34, 1.08)	0.17 (0.06, 0.46)	
*p*_for trend_	0.086	<0.001	
Per SD increment of MTP	0.82 (0.66, 1.02)	0.51 (0.37, 0.70)	
**Model I**			0.004
Q1	1	1	
Q2	0.63 (0.34, 1.17)	0.48 (0.22, 1.05)	
Q3	0.44 (0.23, 0.85)	0.15 (0.05, 0.43)	
Q4	0.46 (0.25, 0.83)	0.09 (0.03, 0.27)	
*p*_for trend_	0.009	<0.001	
Per SD increment of MTP	0.77 (0.62, 0.95)	0.40 (0.28, 0.57)	
**Model II**			0.003
Q1	1	1	
Q2	0.64 (0.34, 1.21)	0.51 (0.23, 1.11)	
Q3	0.43 (0.22, 0.84)	0.17 (0.06, 0.50)	
Q4	0.49 (0.27, 0.91)	0.10 (0.03, 0.31)	
*p*_for trend_	0.018	<0.001	
Per SD increment of MTP	0.81 (0.66, 1.00)	0.42 (0.29, 0.61)	

^a^ Model I were adjusted for maternal age, gestational weeks at time of LFT, gestational weight gain, prepregnancy weight, maternal height, menstrual period and cycle, caesarean delivery, GDM, baby gender, maternal educational and income levels, primiparity, and insomnia, drinking and smoking status before pregnancy based on crude model; model II were further adjusted for maternal serum ALT, AST, γ‐GT, ALP, TBIL, IBIL based on model I.

### 3.3 Associations between the MTP level and gestational duration

Moreover, a significant positive association was identified between the third‐trimester MTP level and gestational duration (Table 4). In full model, each SD increment of third‐trimester MTP corresponded to a 0.13 (0.09, 0.17) weeks increased in gestational duration. As expected, the third‐trimester MTP level also influenced gestational duration in a sex‐specific manner (*p*_for interaction_ = 0.032), and each SD increment of third‐trimester MTP corresponded to a 0.15 (0.09, 0.21) weeks increased in gestational duration in female offspring that was more than that in male offspring [0.11 (0.05, 0.17)].

**TABLE 4:** Association between third‐trimester MTP (per SD) and gestational duration (weeks)^a^

Model	Total	Male offspring	Female offspring	*p*_for interaction_
**Crude model**	0.04 (−0.01, 0.09)	0.00 (−0.06, 0.07)	0.09 (0.02, 0.15)	0.065
**Model I**	0.13 (0.09, 0.18)	0.11 (0.06, 0.17)	0.16 (0.10, 0.22)	0.033
**Model II**	0.13 (0.09, 0.17)	0.11 (0.05, 0.17)	0.15 (0.09, 0.21)	0.032

^a^ Model I were adjusted for maternal age, gestational weeks at time of LFT, gestational weight gain, prepregnancy weight, maternal height, menstrual period and cycle, caesarean delivery, GDM, baby gender, maternal educational and income levels, primiparity, and insomnia, drinking and smoking status before pregnancy based on crude model; model II were further adjusted for maternal serum ALT, AST, γ‐GT, ALP, TBIL, IBIL based on model I.

## 4 DISCUSSION

In this prospective study, we found that nearly all the second‐trimester MTP levels were within the clinical reference range, but more than 40% of the third‐trimester MTP levels were less than the lower limit of normal. Moreover, we found for the first time that the third‐trimester MTP level, not the second‐trimester MTP level, was inversely associated with the risk of PTB and was positively associated with gestational duration. The effects of the third‐trimester MTP on PTB risk and gestational duration were stronger in pregnant women carrying female offspring than those carrying male offspring.

The pregnant women had an adequate second‐trimester MTP status. However, more than 40% of them with their third‐trimester MTP levels less than the lower limit of normal even in urban pregnant women. Although the associations between MTP levels and birth outcomes have rarely been investigated in previous studies, our findings were concordant with one recent case–control study. In line with our findings, a statistically significant lower maternal protein intake (*p* < 0.001) was found in the week preceding the delivery among women who delivered preterm offspring (32 g/d) than that in control women (37.2 g/d), and the maternal protein intake correlated positively with the gestational duration of the offspring (*r* = 0.3, *p* < 0.001) (Awasthi et al., 2015). Likewise, a recent Cochrane review study based on two early randomized controlled trial (RCT) studies involving 449 pregnant women found that pregnant women who received nutritional advice resulting in an increase in protein intake had fewer PTB risk than the control subjects (Ota et al., 2015).

Conversely, in the same Cochrane review study abovementioned based on five RCT studies involving 3,384 pregnant women with balanced energy/protein supplementation found there were no significant effects of balanced energy/protein supplementation on PTB as well as gestational duration. Another RCT study involving 505 pregnant women with high‐protein supplementation also found there were no significant effects of high‐protein supplementation on PTB risk. We also found there was no significant association between protein intake and risk of PTB in our preanalysis (data not showed) which was consistent with the above two RCT studies. As we known, measuring dietary intakes in free‐living human populations is difficult (Willett, 2013). Thus, failure to accurately assess actual dietary protein intake may contribute to the discrepancy. The review itself has concluded that the results should be interpreted with caution. The risk of bias was either unclear or high for at least one category examined in several of the included trials, and the quality of the evidence was low for several important outcomes, such as PTB. Researchers have postulated that different influences of maternal nutrients might exist in male offspring and female offspring (Dearden, Bouret, & Ozanne, 2018; Eriksson, Kajantie, Osmond, Thornburg, & Barker, 2010). Thus, lack of examination of sex‐specific associations in offspring in most of these studies may also explain the discrepancy. Moreover, the effect of the nutritional status or nutritional interventions is very likely to be related to the timing of occurrence during gestation (Harding, 2001). MTP level is known to fall progressively throughout pregnancy (Macdonald & Good, 1971). Therefore, the different timing of exposure assessments may also explain the discrepancy. Importantly, the studies examining protein intake did not exclude the influence of individual differences in digestion and absorption, which may also substantially influence the true relationship between maternal protein status and PTB risk. Finally, different control group settings and sample sizes may also contribute to the discrepancies.

The precise molecular mechanisms underlying the relationship between the third‐trimester MTP level and risk of PTB as well as gestational duration are unclear. Nevertheless, our results are biologically plausible. Infection and inflammation are recognized as major risk factors for PTB (Frey & Klebanoff, 2016; Romero, Dey, & Fisher, 2014). As we known, protein plays important roles in protection against infection and a cascade of inflammatory reactions. A low protein status is found to be associated with immune impairments, which may initiate infection and inflammatory reactions (Welsh et al., 1998; Yamada et al., 2016). Moreover, the serum total protein levels have been found to be significantly lower either in grade‐I, grade‐II or grade‐III protein energy malnutrition than in controls (Rahman & Begum, 2005). Thus, these evidence may support our results. That is a lower total protein may increase the risk of PTB through the initiation of maternal infection and inflammatory reaction. Additionally, uncompensated oxidative stress is also considered as one of important reasons for PTB (Dutta et al., 2016; Feoli et al., 2006; Menon, 2014; Romero et al., 2014; Sultana et al., 2017). Protein malnutrition has been found to be associated with free radical overproduction, which decreases the antioxidant defence system (Feoli et al., 2006; Khare et al., 2014; Sinha, Patro, & Patro, 2018). Thus, a lower maternal protein status that induces oxidative stress may be another possible mechanism implicated in our results. However, more studies are needed to verify these hypotheses.

The strengths of our study are listed below. First, we used uniformly measured MTP level during pregnancy instead of the mother's dietary protein intake to explore the association between the maternal protein status and PTB risk as well as gestational duration, which helped to remove the intrinsic inaccuracy and unreliability of food intake assessment and individual differences in digestion and absorption. Second, we used two time points in the second and third trimesters to explore the associations described above, thus exploring gestational stage‐specific differences. Third, we performed all of our analyses in a sex‐specific manner, helping to identify the sex‐specific differences. Finally, the large sample size and prospective study design allowed examination of the impact of MTP level on the research outcomes.

However, we also acknowledge that several potential limitations exist. First, the LMP of some pregnant women were used the foetal crown‐lump length measured in the first trimester using routine ultrasound examination, which may introduce some potential biases due to the inconsistency of evaluation criteria. In fact, for third‐trimester, there was a 5% (174/3478) of newborns' gestational age were calculated with crown‐lump length measured in the first trimester, and for the second‐trimester, the rate was 4.8% (161/3382). Therefore, we conducted a sensitivity analysis after excluding these data found that these data did not significantly affect our main results (Tables S3 and S4). Second, almost all the second‐trimester MTP levels were within the normal clinical reference range, but more than 40% of third‐trimester MTP levels were under the lower limit of normal. This may lead to the gestational stage‐specific association between maternal TP and PTB risk in our study. Thus, more studies are needed to explore the association between MTP and the risk of PTB. Third, our study population exclusively comprised Han Chinese subjects, and all participants were urban residents. Thus, the generalizability of the observed associations might be limited to similar populations. Fourth, although we adjusted for many potential confounders, we could not completely exclude the possibility of residual confounding by unmeasured factors.

In conclusion, the results of the present study demonstrated that the third‐trimester protein nutrition status was worrying even in urban pregnant women. Trimester‐specific and baby sex‐specific association existed between the protein status and risk of PTB as well as gestational duration. The third‐trimester MTP level was inversely associated with PTB risk and was positively associated with gestational duration. Improving the third‐trimester MTP levels may be helpful for preventing PTB. More studies are needed in the near future to examine the observed associations in other ethnic groups, particularly in undeveloped countries.

## CONFLICTS OF INTEREST

The authors declare no conflict of interest.

## CONTRIBUTIONS

TX, NY and LPH conceived and designed the study; LH, XC, YZ, CZ, YW, QG, MH and XH collected the data. XY, NY and LPH supervised the study conduct; TX and YW contributed in the statistical analysis; TX drafted the manuscript; LPH reviewed and revised the manuscript. All authors have read and approved the final manuscript as submitted.

## Supporting information


**Table S1.** Characteristic of the study subjects according to second‐trimester MTP quartiles ^1^
**Table S2.** Characteristic of the study subjects according to third‐trimester MTP quartiles ^1^
**Table S3.** Association between second‐trimester MTP concentrations (g/L) and risk of PTB (*n* = 3,382) ^1^
**Table S4.** Association between second‐trimester MTP concentrations (g/L) and risk of PTB (*n* = 3,221) ^1^
**Table S5.** Association between third‐trimester MTP concentrations (g/L) and risk of PTB (n = 3,304) ^1^
