# Supplementary material for: Association between the maternal protein nutrition status during pregnancy and the risk of preterm birth
Source: Matern Child Nutr. 2020 Aug 20;17(1):e13043. doi: 10.1111/mcn.13043 (PMC7729649; doi:10.1111/mcn.13043)
Supplement: Supplementary file 1 — Table S1. Characteristic of the study subjects according to second‐trimester MTP quartiles 1 Table S2. Characteristic of the study subjects according to third‐trimester MTP quartiles 1 Table S3. Association between second‐trimester MTP concentrations (g/L) and risk of PTB (n = 3,382) 1 Table S4. Association between second‐trimester MTP concentrations (g/L) and risk of PTB (n = 3,221) 1 Table S5. Association between third‐trimester MTP concentrations (g/L) and risk of PTB (n = 3,304) 1 [file MCN-17-e13043-s001.docx]

**Supplementary Table 1.** Characteristic of the study subjects according to second-trimester MTP quartiles ^1^

|  | **Quartiles of second-trimester MTP concentration, g/L** | | | | ***p* value** |
| --- | --- | --- | --- | --- | --- |
|  | **Q1(42.9-67.6)** | **Q2(67.7-70.2)** | **Q3(70.3-72.8)** | **Q4(72.9-83.1)** |  |
| N | 835 | 863 | 848 | 836 |  |
| Maternal age (years) | 29.38 ± 3.70 | 28.69 ± 3.46 | 28.37 ± 3.15 | 28.09 ± 2.99 | <0.001 |
| Gestational weeks at LFT test | 23.21 ± 4.00 | 21.03 ± 4.43 | 19.51 ± 4.12 | 18.37 ± 3.66 | <0.001 |
| Pre-pregnancy weight (kg) | 53.16 ± 7.41 | 53.24 ± 7.65 | 53.55 ± 7.30 | 53.88 ± 7.93 | 0.196 |
| Maternal height (cm) | 160.03 ± 5.01 | 160.33 ± 5.08 | 160.17 ± 4.95 | 161.03 ± 5.01 | <0.001 |
| Gestational weight gains(kg) | 16.13 ± 5.23 | 16.33 ± 5.49 | 15.92 ± 4.96 | 15.61 ± 5.32 | 0.031 |
| Menstrual period(days) | 5.64 ± 1.20 | 5.67 ± 1.15 | 5.75 ± 1.16 | 5.74 ± 1.16 | 0.114 |
| Menstrual cycle (days) | 30.42 ± 6.52 | 30.13 ± 4.18 | 30.65 ± 5.39 | 30.41 ± 4.36 | 0.225 |
| Cesarean delivery |  |  |  |  | <0.001 |
| Yes | 334 (40.00%) | 328 (38.01%) | 317 (37.38%) | 275 (32.89%) |  |
| No | 385 (46.11%) | 435 (50.41%) | 462 (54.48%) | 474 (56.70%) |  |
| Missing | 116 (13.89%) | 100 (11.59%) | 69 (8.14%) | 87 (10.41%) |  |
| GDM |  |  |  |  | 0.010 |
| Yes | 69 (8.26%) | 77 (8.92%) | 84 (9.91%) | 62 (7.42%) |  |
| No | 750 (89.82%) | 748 (86.67%) | 724 (85.38%) | 732 (87.56%) |  |
| Missing | 16 (1.92%) | 38 (4.40%) | 40 (4.72%) | 42 (5.02%) |  |
| Educational levels (years) |  |  |  |  | 0.852 |
| ≤ 9 | 109 (13.05%) | 121 (14.02%) | 104 (12.26%) | 121 (14.47%) |  |
| 10-15 | 218 (26.11%) | 224 (25.96%) | 221 (26.06%) | 224 (26.79%) |  |
| ≥ 16 | 508 (60.84%) | 518 (60.02%) | 523 (61.67%) | 491 (58.73%) |  |
| Income levels (CNY ^2^) |  |  |  |  | 0.473 |
| ≤ 5000 | 311 (37.25%) | 315 (36.50%) | 311 (36.67%) | 342 (40.91%) |  |
| 5000-9999 | 372 (44.55%) | 394 (45.65%) | 372 (43.87%) | 350 (41.87%) |  |
| ≥ 10000 | 152 (18.20%) | 154 (17.84%) | 165 (19.46%) | 144 (17.22%) |  |
| Primiparity (yes) | 665 (79.64%) | 732 (84.82%) | 747 (88.09%) | 748 (89.47%) | <0.001 |
| Hepatitis B (yes ) | 0(0%) | 0(0%) | 0(0%) | 0(0%) | — |
| Insomnia before pregnancy (yes) | 334 (40.00%) | 333 (38.59%) | 313 (36.91%) | 352 (42.11%) | 0.163 |
| Drinking before pregnancy (yes) | 21 (2.51%) | 11 (1.27%) | 12 (1.42%) | 13 (1.56%) | 0.187 |
| Smoking before pregnancy (yes) | 42 (5.03%) | 27 (3.13%) | 17 (2.00%) | 29 (3.47%) | 0.007 |
| **Biochemical parameters** |  |  |  |  |  |
| ALT (U/L) | 17.59 ± 14.96 | 17.84 ± 13.97 | 20.89 ± 16.32 | 20.22 ± 17.49 | <0.001 |
| AST (U/L) | 18.09 ± 7.59 | 18.54 ± 7.74 | 20.50 ± 10.18 | 20.31 ± 10.31 | <0.001 |
| ALP (U/L) | 53.78 ± 15.82 | 51.44 ± 14.80 | 50.55 ± 14.44 | 50.17 ± 13.69 | <0.001 |
| γ-GT (U/L) | 12.25 ± 6.48 | 13.40 ± 8.61 | 14.71 ± 8.72 | 15.69 ± 8.44 | <0.001 |
| TBIL (umol/L) | 6.04 ± 2.54 | 6.26 ± 3.08 | 6.31 ± 2.66 | 6.52 ± 2.59 | 0.005 |
| DBIL (umol/L) | 2.04 ± 0.98 | 2.25 ± 1.07 | 2.32 ± 1.06 | 2.41 ± 1.01 | <0.001 |
| IBIL (umol/L) | 4.00 ± 2.38 | 3.97 ± 2.36 | 4.01 ± 2.36 | 4.11 ± 2.28 | 0.633 |
| MTP (g/L) | 65.46 ± 1.82 | 69.04 ± 0.75 | 71.51 ± 0.74 | 75.06 ± 1.80 | <0.001 |
| **Outcomes** |  |  |  |  |  |
| Gestational duration(weeks) | 39.45 ± 1.51 | 39.52 ± 1.70 | 39.53 ± 1.42 | 39.49 ± 1.51 | 0.681 |
| PTB (%) | 36 (4.31%) | 35 (4.06%) | 28 (3.30%) | 44 (5.26%) | 0.252 |

^1^Continuous variables were presented as mean ± SD; categorical variables were showed as percentages (%).GDM, gestatinoal diabetes mellitus; ALT, alanine transaminase; AST, aspartate transaminase; ALP, alkaline phosphatase; γ-GT, γ-glutamyl transferase; TBIL, total bilirubin; DBIL, direct bilirubin; IBIL, indirect bilirubin; MTP, maternal plasma total protein; PTB, preterm birth. ^2^ CNY, Chinese Yuan, 1 CNY≈0.13 EUR; 1 CNY ≈0.14 USD.

**Supplementary Table 2.** Characteristic of the study subjects according to third-trimester MTP quartiles ^1^

|  | **Quartiles of third-trimester MTP concentration, g/L** | | | | ***p* value** |
| --- | --- | --- | --- | --- | --- |
|  | **Q1(48.0-63.1)** | **Q2(63.2-65.8)** | **Q3(65.9-68.5)** | **Q4(68.6-82.7)** |  |
| N | 855 | 861 | 870 | 892 |  |
| Maternal age (years) | 28.87 ± 3.52 | 28.62 ± 3.39 | 28.32 ± 3.33 | 28.20 ± 3.44 | <0.001 |
| Gestational weeks at time of LFT | 38.86 ± 2.50 | 38.48 ± 3.00 | 38.08 ± 3.48 | 37.15 ± 4.05 | <0.001 |
| Pre-pregnancy weight (kg) | 53.54 ± 7.29 | 53.45 ± 7.37 | 53.30 ± 7.42 | 53.47 ± 7.58 | 0.925 |
| Maternal height (cm) | 160.26 ± 4.95 | 160.48 ± 5.15 | 160.46 ± 5.04 | 160.32 ± 5.02 | 0.750 |
| Gestational weight gains(kg) | 16.27 ± 4.77 | 16.04 ± 4.79 | 16.26 ± 4.86 | 15.31 ± 5.06 | <0.001 |
| Menstrual period(days) | 5.71 ± 1.20 | 5.70 ± 1.21 | 5.65 ± 1.17 | 5.69 ± 1.20 | 0.785 |
| Menstrual cycle (days) | 30.48 ± 4.69 | 30.35 ± 3.95 | 30.29 ± 4.24 | 30.34 ± 4.39 | 0.812 |
| Cesarean delivery (yes) |  |  |  |  | 0.764 |
| Yes | 325 (38.01%) | 330 (38.33%) | 323 (37.13%) | 349 (39.13%) |  |
| No | 505 (59.06%) | 497 (57.72%) | 509 (58.51%) | 509 (57.06%) |  |
| Missing | 25 (2.92%) | 34 (3.95%) | 38 (4.37%) | 34 (3.81%) |  |
| GDM |  |  |  |  | 0.042 |
| Yes | 87 (10.18%) | 106 (12.31%) | 72 (8.28%) | 78 (8.74%) |  |
| No | 764 (89.36%) | 746 (86.64%) | 794 (91.26%) | 806 (90.36%) |  |
| Missing data | 4 (0.47%) | 9 (1.05%) | 4 (0.46%) | 8 (0.90%) |  |
| Educational levels (years) |  |  |  |  | 0.018 |
| ≤ 9 | 92 (10.76%) | 85 (9.87%) | 102 (11.72%) | 131 (14.69%) |  |
| 10-15 | 210 (24.56%) | 216 (25.09%) | 224 (25.75%) | 242 (27.13%) |  |
| ≥ 16 | 553 (64.68%) | 560 (65.04%) | 544 (62.53%) | 519 (58.18%) |  |
| Income levels (CNY ^2^) |  |  |  |  | 0.530 |
| ≤ 5000 | 298 (34.85%) | 304 (35.31%) | 312 (35.86%) | 317 (35.54%) |  |
| 5000-9999 | 363 (42.46%) | 388 (45.06%) | 373 (42.87%) | 406 (45.52%) |  |
| ≥ 10000 | 194 (22.69%) | 169 (19.63%) | 185 (21.26%) | 169 (18.95%) |  |
| Primiparity (yes) | 697 (81.52%) | 703 (81.65%) | 726 (83.45%) | 762 (85.43%) | 0.098 |
| Hepatitis B (yes ) | 10 (1.17%) | 15 (1.74%) | 16 (1.84%) | 22 (2.47%) | 0.245 |
| Insomnia before pregnancy (yes) | 350 (40.94%) | 355 (41.28%) | 366 (42.07%) | 396 (44.39%) | 0.453 |
| Drinking before pregnancy (yes) | 12 (1.41%) | 12 (1.39%) | 17 (1.95%) | 9 (1.01%) | 0.422 |
| Smoking before pregnancy (yes) | 33 (3.86%) | 25 (2.90%) | 24 (2.76%) | 22 (2.47%) | 0.354 |
| **Biochemical parameters** |  |  |  |  |  |
| ALT (U/L) | 11.28 ± 35.17 | 10.66 ± 15.15 | 11.06 ± 11.91 | 13.39 ± 19.11 | <0.001 |
| AST (U/L) | 16.17 ± 14.76 | 16.53 ± 13.20 | 16.66 ± 9.45 | 18.18 ± 12.68 | <0.001 |
| ALP (U/L) | 159.59 ± 57.54 | 160.12 ± 59.84 | 162.28 ± 74.40 | 156.68 ± 69.02 | 0.142 |
| γ-GT (U/L) | 11.21 ± 8.70 | 11.43 ± 6.64 | 12.35 ± 7.84 | 14.80 ± 11.13 | <0.001 |
| TBIL (umol/L) | 5.50 ± 2.96 | 5.73 ± 2.67 | 6.21 ± 4.02 | 6.48 ± 3.05 | <0.001 |
| DBIL (umol/L) | 2.40 ± 1.55 | 2.37 ± 1.01 | 2.48 ± 1.19 | 2.43 ± 1.25 | 0.230 |
| IBIL (umol/L) | 3.10 ± 2.16 | 3.44 ± 3.45 | 3.64 ± 2.49 | 4.05 ± 2.62 | <0.001 |
| MTP (g/L) | 60.70 ± 2.09 | 64.56 ± 0.79 | 67.15 ± 0.78 | 71.15 ± 2.20 | <0.001 |
| **Outcomes** |  |  |  |  |  |
| Gestational duration(weeks) | 39.51 ± 1.49 | 39.56 ± 1.39 | 39.68 ± 1.30 | 39.59 ± 1.31 | 0.078 |
| PTB (%) | 43 (5.03%) | 29 (3.37%) | 20 (2.30%) | 28 (3.14%) | 0.017 |

^1^ Continuous variables were presented as mean ± SD; categorical variables were showed as percentages (%).GDM, gestatinoal diabetes mellitus; ALT, alanine transaminase; AST, aspartate transaminase; ALP, alkaline phosphatase; γ-GT, γ-glutamyl transferase; TBIL, total bilirubin; DBIL, direct bilirubin; IBIL, indirect bilirubin; MTP, maternal plasma total protein; PTB, preterm birth. ^2^ CNY, Chinese Yuan, 1 CNY≈0.13 EUR; 1 CNY ≈0.14 USD.

**Supplementary Table 3.** Association between second-trimester MTP concentrations (g/L) and risk of PTB (n=3382) ^1^

|  | **Quartiles of second-trimester MTP concentration, g/L** | | | | ***p* for trend** | **Per SD increment of MTP** |
| --- | --- | --- | --- | --- | --- | --- |
|  | **Q1**  **(42.9-67.6)** | **Q2**  **(67.7-70.2)** | **Q3**  **(70.3-72.8)** | **Q4**  **(72.9-83.1)** |  |  |
| Crude model | 1 | 0.71 (0.44, 1.13) | 0.49 (0.30, 0.81) | 0.71 (0.45, 1.11) | 0.122 | 0.92 (0.78, 1.10) |
| Model 1 | 1 | 0.95 (0.59, 1.54) | 0.80 (0.47, 1.35) | 1.25 (0.76, 2.06) | 0.422 | 1.17 (0.98, 1.41) |
| Model 2 | 1 | 0.92 (0.57, 1.49) | 0.75 (0.44, 1.28) | 1.16 (0.71, 1.90) | 0.616 | 1.13 (0.94, 1.36) |

^1^ Model I were adjusted for maternal age, gestational weeks at time of LFT, gestational weight gain, pre-pregnancy weight, maternal height, menstrual period and cycle, cesarean delivery, GDM, baby gender, maternal educational and income levels, primiparity, and insomnia, drinking and smoking status before pregnancy based on crude model; Model II were further adjusted for maternal serum ALT, AST, γ-GT, ALP, TBIL, IBIL based on model I.

**Supplementary Table4.** Association between second-trimester MTP concentrations (g/L) and risk of PTB (n=3221) ^1^

|  | **Quartiles of second-trimester MTP concentration, g/L** | | | | ***p* for trend** | **Per SD increment of MTP** |
| --- | --- | --- | --- | --- | --- | --- |
|  | **Q1** | **Q2** | **Q3** | **Q4** |  |  |
| Crude model | 1 | 0.70 (0.44, 1.13) | 0.48 (0.29, 0.80) | 0.70 (0.44, 1.10) | 0.120 | 0.90 (0.76, 1.08) |
| Model 1 | 1 | 0.94 (0.58, 1.52) | 0.77 (0.45, 1.31) | 1.22 (0.74, 2.03) | 0.496 | 1.14 (0.94, 1.38) |
| Model 2 | 1 | 0.92 (0.56, 1.50) | 0.72 (0.42, 1.23) | 1.13 (0.68, 1.88) | 0.717 | 1.09 (0.90, 1.32) |

^1^ Excluding 4.8% (161/3382) of newborns’ gestational age were calculated with crown-lump length measured in the ﬁrst trimester. Model I were adjusted for maternal age, gestational weeks at time of LFT, gestational weight gain, pre-pregnancy weight, maternal height, menstrual period and cycle, cesarean delivery, GDM, baby gender, maternal educational and income levels, primiparity, and insomnia, drinking and smoking status before pregnancy based on crude model; Model II were further adjusted for maternal serum ALT, AST, γ-GT, ALP, TBIL, IBIL based on model I.

**Supplementary Table5.** Association between third-trimester MTP concentrations (g/L) and risk of PTB (n=3304) ^1^

|  | **Quartiles of third-trimester MTP concentration, g/L** | | | | ***p* for trend** | **Per SD increment of MTP** |
| --- | --- | --- | --- | --- | --- | --- |
|  | **Q1(48.0-63.1)** | **Q2(63.2-65.8)** | **Q3(65.9-68.5)** | **Q4(68.6-82.7)** |  |  |
| **Crude model** | 1 | 0.59 (0.37, 0.95) | 0.35 (0.20, 0.61) | 0.39 (0.24, 0.65) | <0.001 | 0.69 (0.57, 0.83) |
| **Model I** | 1 | 0.59 (0.36, 0.96) | 0.33 (0.19, 0.58) | 0.29 (0.17, 0.48) | <0.001 | 0.64 (0.53, 0.77) |
| **Model II** | 1 | 0.61 (0.37, 0.99) | 0.35 (0.20, 0.62) | 0.30 (0.18, 0.52) | <0.001 | 0.66 (0.55, 0.80) |

^1^ Excluding 5% (174/3478) of newborns’ gestational age were calculated with crown-lump length measured in the ﬁrst trimester. Model I were adjusted for maternal age, gestational weeks at time of LFT, gestational weight gain, pre-pregnancy weight, maternal height, menstrual period and cycle, cesarean delivery, GDM, baby gender, maternal educational and income levels, primiparity, and insomnia, drinking and smoking status before pregnancy based on crude model; Model II were further adjusted for maternal serum ALT, AST, γ-GT, ALP, TBIL, IBIL based on model I.
